# Synergistic Effect and Molecular Mechanisms of Traditional Chinese Medicine on Regulating Tumor Microenvironment and Cancer Cells

**DOI:** 10.1155/2016/1490738

**Published:** 2016-03-02

**Authors:** Jingnan Xu, Zhuo Song, Qiujun Guo, Jie Li

**Affiliations:** ^1^Department of Oncology, Guang'anmen Hospital, China Academy of Chinese Medical Sciences, No. 5 Beixiange, Xicheng District, Beijing 100053, China; ^2^Beijing University of Chinese Medicine, No. 11 North Third Ring Road East, Chaoyang District, Beijing 100029, China; ^3^China Academy of Chinese Medical Sciences, No. 16 Nanxiaojie Dongzhimennei, Dongcheng District, Beijing 100700, China

## Abstract

The interaction of tumor cells with the microenvironment is like a relationship between the “seeds” and “soil,” which is a hotspot in recent cancer research. Targeting at tumor microenvironment as well as tumor cells has become a new strategy for cancer treatment. Conventional cancer treatments mostly focused on single targets or single mechanism (the seeds or part of the soil); few researches intervened in the whole tumor microenvironment and achieved ideal therapeutic effect as expected. Traditional Chinese medicine displays a broad range of biological effects, and increasing evidence has shown that it may relate with synergistic effect on regulating tumor microenvironment and cancer cells. Based on literature review and our previous studies, we summarize the synergistic effect and the molecular mechanisms of traditional Chinese medicine on regulating tumor microenvironment and cancer cells.

## 1. Introduction

Tumor microenvironment (TME) plays a pivotal role in the process of cancer development and metastasis. Tumor and its microenvironment are a complex compound including the “seed” and “soil,” which was first proposed by Stephen Paget; a hypothesis suggested that the sites where metastases occur were defined not only by the tumor cell (seed) but also by the microenvironment of the secondary metastatic site (soil) [1]. Until recently, the staging and treatment approaches for cancer appeared to be orientated predominantly to both “soil” and “seed.” We are currently witnessing an increasing amount of evidence, spanning from clinical to laboratory research, which highlights that cancer growth and metastasis are the result of the dynamic balance between the cancer itself and the impaired function of the TME [2]. Target organs can release various cytokines recruiting tumor cells, promoting cell proliferation, and inducing angiogenesis and eventually form metastases. At the same time, tumor cells can also release various cytokines by paracrine manner, remodeling TME for their own survival. Therefore, the interaction between tumor cells and TME of target organ facilitates a complex metastasis process. Increasing evidence indicates that TME is a key target of tumor therapy research, because of its special physical and chemical properties and the internal relations between inflammation and immune system [3]. The final purpose of target therapy on TME is effectively resisting the interaction between tumor cells and their microenvironment. However, conventional cancer treatments mostly focused on tumor cell and single targets or single mechanism, on the basis of the fact that few researches intervene in the whole TME [4]. In recent years, researches of antitumor drugs focused on remodeling TME emerge endlessly, mainly targeting antiangiogenesis and immunotherapy to overcome the immune tolerance, treatment reversing drug resistance, and so forth. Unexpectedly, antiangiogenesis drugs did not achieve the ideal treatment effect; on the contrary they produce greater toxicity and promote the development of tumor as a result of hypoxia and reduction of transmission chemotherapy drugs to tumor tissue, promoting tumor drug resistance; tumor cells can get nutrition energy supplement through autophagy from TME and eventually make the antiangiogenesis therapy only show decreasing repair ability of normal tissue [5]. Tumor immunotherapy has become an important means to prevent tumor recurrence and metastasis. Most current tumor immunotherapy has shown good effect of tumor destruction in vitro but did not reach expected effect in vivo, for it cannot overcome the tumor antigen-presenting and immune effectors function inefficiency. Conventional tumor chemotherapy resistance research mainly focused on the genetic changes of endogenous factors. A large number of studies have shown that the TME played an important role in mediating acquired drug resistance [6].

In cancer treatment, traditional Chinese medicine (TCM) emphasizes the overall efficacy, inhibiting tumor cell as well as TME to suppress tumor development and recurrence. So far, many Chinese herbs have been shown to have a good effect in clinical studies, which display a broad range of clinical effects including alleviation of cancer-associated symptoms, prolonging survival rates, decreasing treatment-related toxicity, and preventing recurrence and metastasis [7–15], as shown in Table 1. Furthermore, several Chinese herbs have also been proven to inhibit tumors in fundamental experiments. Although the mechanism of TCM is still unclear, increasing evidence has shown that it may relate with synergistic effect on regulating TME and cancer cells. In this review, we will summarize the synergistic effect and the molecular mechanisms of TCM on regulating TME and cancer cells according to recent researches.

## 2. TCM Regulates Tumor Microenvironment

The TME encompasses a complex meshwork of nonmalignant cells, structural components, molecules, and chemicals that surround cancer cells. The nonmalignant cells, including endothelial cells, pericytes, fibroblasts, and immune cells, together with the surrounding extracellular matrix (ECM), comprise the supportive stroma of the tumor and modulate the TME [16]. The production of both tumor-promoting and tumor-suppressing signals from these various cell types influences the tumor microenvironment. Recently, targeting TME has opened new avenues in clinical oncology [17]. However, treatment itself activates the microenvironment by damaging a large population of cells, which can drastically exacerbate disease conditions in a cell in a nonautonomous manner, and such off-target effects should be well taken into account when establishing future therapeutic rationale [18]. TCM is a potential treatment strategy.

### 2.1. TCM Inhibits the Degradation of Extracellular Matrix

The tumor microenvironment consists of an insoluble ECM, a stroma composed of fibroblasts, adipocytes, and endothelial and resident immune cells, and a multitude of growth factors and cytokines [19]. Abnormal changes in the amount and organization of molecules lead to altered biochemical and physical properties of tumor-associated ECM that contributes to tumor progression and resistance to therapy [20, 21]. Matrix metalloproteinases (MMPs) are enzymes that degrade structural components of the ECM, produced by cancer-associated fibroblasts (CAFs), tumor-associated macrophages (TAMs), tumor-associated neutrophils (TAN), mast cells (MCs), blood endothelial cells (BECs), lymphatic endothelial cells (LECs), and bone marrow-derived mesenchymal stem cells (MSCs). These enzymes regulate a multitude of physiological processes such as morphogenesis, tissue remodeling, and signaling events [22]. Its activity has been implicated in almost every stage of the metastatic cascade from the primary site to the progression of tumor extravasation, growth, and development. The expression and activity of MMPs against matrix macromolecules have been associated with the development of malignant phenotypes and the promotion of cell invasiveness and metastasis [23]. Several studies show that MMP-2 and MMP-9 are highly expressed in tumors and are associated with poor clinical outcome. Some traditional Chinese medicine monomers and compounds have been reported to have inhibitory effects on the migration and invasion of cancer cells via reducing the expression of MMPs [24, 25].

Deng et al. illustrated YQFS (a standard formulation of Si-Jun-Zi-Tang with the addition of* Myristica fragrans* and five-leaf* Akebia* fruit) extract had an antitumor effect, which could be attributed to ERK1/2-dependent inhibition of MMP-2/9 expression, modulating the ERK/MAPK pathway and its downstream factors by selectively targeting ERK phosphorylation [24].* Momordica cochinchinensis* (a Chinese herbal called Mu BieZi) has been used for a variety of purposes, showing an anticancer action. Zheng et al. found that extracts of* Momordica cochinchinensis* seeds (ESMCs) revealed strong growth inhibitory effects on ZR-75-30 cells and effectively inhibit ZR-75-30 cell invasion in a dose-dependent manner. ESMC treatment could not only reduce the protein expression but also repress the enzymatic activity of MMP-2 and MMP-9, which suggests that ESMC's anti-invasive action was mediated by diminishing the ability of breast cancer cells to degrade the components of ECM by modulating MMP-2 and MMP-9 expression and activity [26].

### 2.2. TCM Improves the Hypoxia Microenvironment

Abnormal and dysfunctional blood vessels in tumor tissues are incapable of restoring oxygenation, therefore perpetuating hypoxia, which, in turn, will fuel tumor progression, metastasis, and resistance to antitumor therapies [27]. Increasing evidences indicate that the vasculature is insufficient to supply adequate oxygen when solid tumor diameter is >2 mm, resulting in local hypoxic and anoxic conditions inside the tumor. The level of hypoxia within a tumor increases during tumor progression and is a good indicator of disease outcome because hypoxia selects the most invasive cancer cells and promotes resistance to therapies [28–30]. Factors in the response of tumor cells to this distinct microenvironment are the activities of the hypoxia inducible factor-1*α* (HIF-1*α*), which is regulated in an oxygen-dependent manner [31]. HIF-1*α* signaling pathway is frequently observed in solid tumors and is strongly associated with numerous pathophysiological processes, including the induction of epithelial-mesenchymal transition (EMT), a process in which epithelial cells lose cell-cell adhesion and cell polarity and acquire properties of mesenchymal cells, which results in cancer progression, metastasis, and multidrug resistance in cancer [32–35].

Pien Tze Huang (PZH) has been used in China and Southeast Asia for centuries as a remedy for various types of human cancer. It was found that treatment with PZH was observed to significantly decrease the cell migration and invasion rates and inhibit the hypoxia-mediated EMT and HIF-1 signaling, suggesting that PZH concentration dependently inhibits the hypoxia-induced metastasis of colon cancer cells [35]. Another study showed that Oroxylin A remarkably inhibited HIF-1*α* expression and its stability and also suppressed the downstream targets (e.g., PDK1, LDHA, and HK II) and their mRNA levels under hypoxia. Furthermore, Oroxylin A could decrease the accumulation of ROS, which was benefit for inhibition on glycolytic activity by decreasing ROS-mediated HIF-1 expression. PI3K is an important molecular signal transduction in tumor cells and an upstream signal pathway molecular of HIF-1*α* [36]. Wogonin, extracted from the TCM herb* Scutellaria baicalensis*, was recently found to be a good candidate for the development of new multidrug resistance (MDR) reversal agent and its reversal mechanism was due to the suppression of HIF-1*α* expression via inhibiting PI3K/Akt signaling pathway [37, 38].

### 2.3. TCM Reverses the Immunosuppressive Microenvironment

Suppressive phenotypes of immune cells are regulated and reversed by TCM treatment. Concretely, inflammatory T cells and natural killer T cells (NKT) increasingly proliferate in contrast of the reducing quantity of regulatory T cells and myeloid-derived suppressor cells (MDSCs) after treatment by TCM. Furthermore, suppressive macrophages and dendritic cells (DCs) change their functions to antitumor effects, such as M2 to M1 phenotype reversing and DCs maturation by increasing inflammatory factors expression and immune suppressive cytokines decreasing [16].

The major function of DCs is to process and present antigen for the activation of CD4^+^ and CD8^+^ T cells. Endocytosis of antigen by immature DCs drives DCs maturation and the subsequent presentation of antigen to T cells [39]. However, the tumor microenvironment systemically perturbs this process by increasing the accumulation of immature DCs and decreasing DCs maturation. As a result, DCs fail to activate T cells. Defective DCs function has been found in many patients with a variety of cancers, such as pancreatic carcinoma, cervical squamous intraepithelial lesions, hepatocellular carcinoma, and non-small cell lung cancer [39–43]. Multiple conditions and factors within the TME cause DCs abnormalities, including hypoxia, lactic acid build-up, and adenosine accumulation.* Astragalus mongholicus* (AMs) is a common herbal of TCM and has been proved to be effective in treating cancers according to abundant clinical case reports. Tian et al. investigated the effect and mechanism of AMs on human stomach cancer. It turned out that AMs is effective in treating stomach cancer and it might precipitate DCs maturation by regulating TLR4 mediated NF-*κ*B signal transduction against tumor [44].

MDSCs are a major host component contributing to the immune suppressive environment, inhibiting both adaptive and innate antitumor immunity through different ways, such as inhibiting T cell activation and function and suppressing natural killer cell (NK) cytotoxicity [16]. MDSCs represent a heterogeneous population, including immature macrophages, DCs, and granulocytes, generated by and released from the bone marrow in response to a wide array of pathological stimulations like malignant tumor and inflammation, leading to the expansion of MDSCs and contributing to the negative regulation of tumor immune response [45]. The MDSCs can inhibit T cell-mediated tumor acquired immune responses with overexpression of Arg1, iNOS, and Ros, suppress NK cell cytotoxicity by inhibiting NKG2D and IFN-c, and facilitate angiogenesis by releasing vascular endothelial growth factor (VEGF), MMPs, and TGF-*β* [46]. Wang et al. investigated the effect of ginseng-derived compound K (C-K) on apoptosis, immunosuppressive activity, and proinflammatory cytokine production of MDSCs. It turned out that C-K treatment can significantly increase the percentages of early and late apoptotic MDSCs in vitro, decrease the expressions of immunosuppression-related genes Cox-2 and Arg-1, and suppress the function of IL-1*β*, IL-6, and IL-17, which implied that C-K can restrain the immunosuppressive effect of MDSCs to inhibit tumor cell proliferation in mice [47].

Tumor-associated macrophages (TAMs) are a heterogeneous population of myeloid cells with a potential to promote cancer cell proliferation and invasion and regulate tumor neovascularization and lymphangiogenesis, as well as cytotoxic T cell function [47]. Under pathological conditions, macrophages acquire distinct phenotypic characteristics through different activation mechanisms [48]. The classical activated macrophage (M1-like) exhibits proinflammatory properties by expressing and secreting proinflammatory molecules (e.g., TNF-*α*, IL-6, IL-12, IL-1, Type I, IFN-*γ*, CXCL1–3, CXCL-5, and CXCL8–10), while macrophage could be alternatively activated to M2-like phenotype, which expresses anti-inflammatory factors (e.g., VEGFC/D, VEGFR3, MMPs, IL-10, and IL-13) [49–52]. Several researchers have reported TCM could switch the phenotype of TAMs from M2 to M1, inhibiting tumor progression. Baicalin is a natural flavonoid from medicinal herbs including* Scutellaria baicalensis* Georgi. Several studies have revealed the antitumor action of Baicalin by increased expressions of IFN-*γ* and IL-12 to activate immune response. Recent study results showed that Baicalin can initiate TAM reprogramming to M1-like macrophage, induce repolarization of TAM and M2-like macrophage through autophagy and transcriptional activation of RelB/p52 pathway, and promote proinflammatory cytokines production [53, 54].

### 2.4. TCM Inhibits Angiogenesis

The growth and metastasis of the tumor depend on an effective microcirculation. The formation of a microcirculation can occur via the traditionally recognized mechanisms of angiogenesis and vasculogenic mimicry (VM). It is well established that angiogenesis is necessary for the tumor progression and metastasis [55]. Angiogenesis occurs by complex sequential steps, such as basement membrane degradation by proteases, endothelial cell proliferation and migration/invasion, formation of capillary tubes, and survival of newly formed blood vessels, which is tightly regulated by an intricate balance between stimulators and inhibitors such as VEGF, fibroblast growth factor, and MMPs [56]. Among them, VEGF is the most important angiogenic factor closely associated with neovascularization in human tumors [57]. VM is a novel tumor blood supply in some highly aggressive malignant tumors, provides a special passage without endothelial cells, and is conspicuously different from vasculogenesis [58]. VM has unique ability of highly aggressive tumor cells to express endothelial cell-associated genes and form ECM-rich, patterned tubular networks when cultured on a three-dimensional (3D) matrix and is associated with a poor prognosis for the patients with some aggressive malignant tumors [59, 60].

Ginsenoside Rg3, a saponin extracted from ginseng, has been demonstrated to have anticancer activity in vitro and in vivo with relatively low toxicity, especially on vessels or angiogenesis in tumors [61]. A study aimed to investigate the antiangiogenic effects of Rg3 in patients with acute leukemia. The results showed that Rg3 exhibited antileukemia effect in part due to its antiangiogenic activity via inhibiting PI3K/Akt and ERK1/2 pathways, which act to regulate the expression of HIF-1*α* and VEGF [62]. The rhizome of* Atractylodes lancea* is extensively used in Chinese medicine as crude extracts/decoctions or a component in various herbal formulations. Numerous studies have reported the anticancer activities of* Atractylodes lancea* [63]. CLT, also named Atractylenolide III, is the major bioactive component of* Atractylodes lancea*. Wang et al. demonstrated CLT inhibited the development of angiogenesis both in vitro and in vivo. Similar to its effects on cancer cells, the inhibitory effect of CLT on ECs was due to its ability to inhibit MMPs expression and VEGF secretion by downregulating Runx2 activation of ECs, which may be associated with interference with BMP signaling in endothelial cells [64]. Abundant studies have demonstrated that PZH could suppress multiple colorectal cancers and associated signaling pathways, leading to the promotion of cancer cell apoptosis and the inhibition of cell proliferation and tumor angiogenesis [65–70].

Most antiangiogenic therapies currently being evaluated target the VEGF pathway. However, the tumor vasculature can acquire resistance to VEGF-targeted therapy by shifting to other angiogenesis mechanisms. Therefore, other therapeutic agents that block non-VEGF angiogenic pathways need to be evaluated. Recent studies have identified fibroblast growth factor 1 (FGF1) as a direct activator of PI3K-Akt, which is a non-VEGF angiogenic pathway to initiate endothelial cell migration, invasion, and differentiation. Ferulic acid (FA), an effective component of many Chinese medicinal herbs, like* Cimicifuga heracleifolia*,* Angelica sinensis*, and* Lignsticum chuangxiong*, exhibits anti-inflammatory and anticancer activities. A study indicated that FA exerted antiangiogenesis activities at a nontoxic dosage via specifically targeting fibroblast growth factor receptor 1 (FGFR1) and its PI3K/Akt signaling pathway in melanoma [71].

### 2.5. TCM Inhibits Lymphangiogenesis

There is accumulating evidence that tumor-associated lymphangiogenesis is an important feature of tumor progression and may facilitate cancer cell dissemination to the lymph nodes [72, 73]. Accordingly, numerous clinical studies have demonstrated a significant correlation between lymphatic vessel density and lymph node metastasis. Clinical evidence suggests that the VEGF family members VEGF-C and VEGF-D are major lymphangiogenic regulators by binding to VEGFR-2 and VEGFR-3, which are expressed on LECs. Higher VEGF-C expression is associated with higher peritumoral lymphatic vessel density, increased lymphatic invasion, and increased lymph node metastasis [74]. Kimura and Sumiyoshi discovered Wogonin isolated from* Scutellaria baicalensis* roots could inhibit VEGF-C-induced lymphangiogenesis through a reduction in VEGF-C-induced VEGFR-3 phosphorylation [75]. Norcantharidin (NCTD) is a demethylated and low-cytotoxic derivative of cantharidin with antitumor properties, an active ingredient of the traditional Chinese medicine* Mylabris*. It has been reported that NCTD is used selectively in clinic to treat hepatic, gastric, colorectal, and ovarian carcinomas and leucopenia in China because of its effective anticancer activity, fewer side effects, and leukocytosis [76–81]. A study showed that NCTD inhibited tumor growth and lymphangiogenesis of HCACs through “multipoints priming” mechanisms, that is, directly or indirectly downregulating VEGF-A, -C, -D/VEGFR-2, -3 signaling pathways, which strongly suggested that NCTD could serve as a potential antilymphangiogenic agent for tumor lymphangiogenesis [82]. Nagy et al. demonstrated that, in addition to angiogenesis, VEGF-A also induced proliferation of lymphatic endothelium, resulting in the formation of greatly enlarged and poorly functioning lymphatic channels, and abnormal lymphangiogenesis. These findings raise the possibility that abnormal lymphangiogenesis may also be expected in other circumstances such as malignant tumors characterized by VEGF-A overexpression [81].

## 3. TCM Inhibits the Development of the “Seed”

Just as the role of a seed in growth, cancer cells play a pivotal role in the process of tumor occurrences, developments, and metastasis, which are always the focus and hot areas of tumor researches. It was reported that this “Seed” can proliferate immortally and activate invasions and metastasis [83]. In addition, tumor cells were also deemed to be the initiators of tumor microenvironment formation, which reversely promote the growth of tumor cells [84]. Nowadays, surgery, radiotherapy, and chemotherapy are three major treating strategies of tumors for minishing burden and inhibiting growth, which have been widely used and achieved effects in clinic [85]. However, these targeted treatments were reported to have some degree of adverse effect with them. TCM, as an important complementary strategy, has also been shown to possess therapeutic effects on cancer cells. The followings may be the principle and potential molecular mechanisms for TCM in cancer treatment.

### 3.1. TCM Inhibits the Growth of Cancer Cells

Proliferation and apoptosis of cells are two critical factors that determine organism development and tissue homeostasis [86, 87]. Precisely, it is the unrestricted proliferation and suppressed apoptosis that make cancer cells grow frantically and malignantly. Accumulating studies have revealed that dysfunction in cell cycle regulation often resulted in abnormal proliferation of cancer cells [88]. The regulation of the cell cycle is influenced by many molecules such as cyclins, CDKs, and CDKIs and through Akt and MAPK signaling pathways [89]. In addition, p53, a major mediator of cell cycle arrest, was reported to mutate or be inactivated in several tumors. If that happened, the apoptotic response is not activated and cell proliferation is allowed [90]. Studies have shown that p53 can upregulate the level of p21 and Gadd45 to mediated cell cycle arrest [91–95]. Others also implicated that p53 upregulated proapoptotic Bax and downregulated prosurvival Bcl-2 to mediate apoptosis [96, 97]. Several mechanisms also demonstrated that overexpression of Bcl-2 increases the activity of AKT and IKK as well as NF-*κ*B transcriptional activity in cancer [98]. TCM has been reported to inhibit the growth of cancer cells in abundant clinical trials. Although the mechanisms are not very clear, increasing data has shown that they maybe relate with above-mentioned regulating biomarkers to inhibit proliferation and induce apoptosis of cancer cells. The confirmed mechanisms of herb compounds and monomers acting common cancers are listed in Table 2 [86, 88, 91, 92, 95, 97, 99–103].

### 3.2. TCM Prevents Invasion and Metastasis of Tumor Cells

Despite the fact that all available treatments had been implemented, local invasion and metastasis predict a poor prognosis and contribute to more than 90% of tumor mortality [104, 105]. Therefore, it is needed to gain further insight into the molecular mechanisms on invasion and metastasis of tumor cells and to search for more effective therapies. Extracellular matrix (ECM) is related to the tumor cell invasion and metastasis, and, as we know, the abnormal and absent expression of ECM has been found in many malignant cells [106]. In tumor microenvironment, tumor cells can degrade and remodel the ECM by excessively secreting matrix MMPs such as MMP-2 and MMP-9 [107]. In the meantime tumor cells also conducted epithelial-to-mesenchymal transition (EMT) process, downregulating E-cadherin expression and overexpressing N-cadherin and vimentin [108, 109], which weakens intercellular adhesive attractions. In addition, increased levels of phosphorylated p38*α* could downregulate fibulin-3 expression through hypermethylation of regulatory sequences of the gene and then facilitated the invasion and metastasis of tumor cells [110].

TCM can prevent invasion and metastasis of tumor cells via inhibiting ECM degradation and EMT process. Fei-Liu-Ping (FLP) ointment is an oral prescription medication that is used to treat lung cancer patients in China, which has been shown to possess anticancer properties [64]. Li et al. revealed that FLP could inhibit A549 cell invasion and metastasis by increasing E-cadherin expression and decreasing the expression of N-cadherin and MMP9. They also found that FLP performed synergistic effect when combined with cyclophosphamide (CTX) [111]. Baicalein, another antineoplastic compound of Chinese herbs, also inhibited the expression of MMP-9 and MMP-2 via reducing expression of protein kinase C*α* (PKC*α*) and p38 mitogen-activated protein kinase (p38 MAPK) levels in poorly differentiated hepatoma cells [112]. The anti-invasive and antimigratory effects of TCM were also found in curcumin researches. Curcumin can prevent the invasion of hepatocellular carcinoma through inhibiting the production of MMP-9. In addition, it was found that curcumin significantly inhibited adhesion and haptotactic migration to fibronectin and laminin without affecting the expression of integrin on the cell surface. Furthermore, it could also affect the formation of actin stress fibers [113].

### 3.3. TCM Reverses the Immunosuppressive Phenotype of Tumor Cells

As the immune-editing theory says, immune system plays a critical role in maintaining equilibrium between immune recognition and tumor development, with a dual capacity to both promoting and suppressing tumor growth [114]. During cancer immune editing, the immune system is able to recognize and destroy the most immunologically vulnerable cancer cells. Nonetheless, due to genetic instability, constant tumor cell division generates with reduced immunogenicity that can evade immune elimination [115]. Furthermore, tumor cells can also vary several immune phenotypes to impair the capacity of the immune system to eradicate them by immune suppressive effects [116]. In the recent years, studies have shown that, while being treated by TCM, the expression of programmed cell death protein-1 (PD-1) was decreased. Classic major histocompatibility complex (MHC) and Fas molecules were expressed more on tumor cytomembranes, which leaded tumor cells to be recognized easier and killed by the immune system. In conclusion, TCM can exert a biphasic regulation on tumor cells phenotype to enhance antitumor immune responses.

#### 3.3.1. TCM Promotes Classic MHC Molecules Expression

MHC is the important immunological recognition molecule in the process of tumor immune response. Classic MHC molecule can be divided into two subgroups: MHC I and MHC II; both of them reinforce the interactions of cytotoxic T cell (CTL) or NK cells with tumor cells by presenting tumor antigens to them [117]. However, immune and malignant cells in the tumor microenvironment do not express typical MHC molecules but overexpress sHLA and sNKAL, which may lead to killing CTL and NK cell mediated by apoptosis [118]. Increasing data has shown that TCM can efficiently reverse this harmful phenomenon. Li et al. explored the effect of Invigorating Spleen and Detoxification Decoction (ISD) (*Radix Codonopsis*,* Poria*,* Rhizoma Atractylodis Macrocephalae*,* Radix Glycyrrhizae*,* Radix Bupleuri*,* Rhizoma Curcumae*, and herba* Scutellaria barbata*) on MHC molecules in the rat liver cancer tissue and found that ISD could enhance the expression of MHC I and MHC II both in tumor and in liver tissue, besides prolonging the survival time and decreasing the incidence of cachexia [119].

#### 3.3.2. TCM Reverses the FasL/Fas Expression of Tumor Cells

FasL and its receptor Fas are membrane-bound glycoproteins; the activation of them plays an important role in cell apoptosis. Physiologically, cytotoxic T lymphocytes (Fas^low^FasL^high^) express FasL combined with the Fas expressing cells (Fas^high^  FasL^low^), resulting in the activation of the Fas receptor, and then mediate target cells apoptosis [120]. However, loss of Fas and gain of aberrant FasL expression are common features of malignant transformation, such as FasL and sFasL, which in turn combined with Fas express lymphocytes and eliminated their activated immune reactions [121]. Our previous study showed that TCM formula Yang Wei Kang Liu (YWKL) (*Radix Astragali*,* Radix Ginseng*,* Hedyotis diffusa*,* Yunnan Manyleaf Paris Rhizome*,* Radix Notoginseng*,* Radix Paeoniae Rubra*, and* Hematoxylon*) could increase Fas expression, downregulate FasL-mRNA expression in MGC-803 stomach cancer cell model in vitro, and induce the apoptosis of MGC-803 cells. Together with the previous research, it indicated that potential mechanisms of YWKL inducing gastric cancer cells apoptosis might be through regulating Fas/FasL pathway so as to enhance cancer cells' sensitivity to immune response cells like CTL [122].

#### 3.3.3. TCM Decreases the Expression of PD-L1

PD-1, which is expressed on activated T and B cells, natural killer cells, and myeloid cells, is an another immune checkpoint [123]. Two ligands for PD-1 have been identified: PD-L1 and PD-L2. Researches indicated that PD-L1 was overexpressed by various tumor cells, including breast cancer, thyroid carcinomas, lung, colon, ovarian, melanoma, bladder, liver, salivary, stomach, and gliomas [124]. PD-L1 can engage the PD-1 receptor and induce T cell exhaustion and eventually inhibit T cell activation and proliferation. Thus, the interaction between PD-1 and its ligands, especially PD-L1, may contribute to the immune evasion of cancer cells [124]. Recently, Wang et al. found that* Astragalus* polysaccharide (APS), extracted from TCM herb* Astragalus*, could significantly inhibit the growth of B16-F10 melanoma cells in a transplant model and decreased the expression of both PD-L1 protein and PD-L1 mRNA in tumor. This indicated that mechanism may be related to regulating PD-1/PD-L1 pathway to enhance the antitumor immune activity of T lymphocytes [125].

### 3.4. TCM Reverses the Drug Resistance of Tumor Cells

Over the past few decades, the efficiency of endless chemotherapies did not reach what we have expected. Some experts attributed this phenomenon to the multidrug resistance (MDR) [126]. It was reported that major mechanical of MDR in tumor cells was the overexpression of a membrane-bound protein, P-glycoprotein (P-gp), and other members of the adenosine triphosphate (ATP) binding cassette (ABC) transporter superfamily [127], which translocate a substrate from the intracellular compartment to the extracellular compartment, leading to a reduced intracellular concentration of the substrate and resistance to antineoplastic drugs [128]. However, several other mechanisms are also involved in the development of MDR in tumor cells, including alterations in drug targets, the activation of detoxifying systems, the interruption of signaling pathways, and alterations in regulators involved in cell cycle control [128].

Recently, accumulated basic researches have proven that TCM could reverse multidrug resistance of tumor cells through several pathways. Yiqi Jianpi Huaji Decoction (YJHD), a traditional Chinese medicinal formula composed of twelve ingredients, has recently been reported to have a good clinical therapeutic effect. Li et al. found that low dose YJHD could reverse MDR and increase sensitivity of cancer cells to chemotherapeutic agents in vitro by downregulating P-gp, MRP, TUBB3, and STMN1 expression [129]. MDR can also be reversed by siRNAs targeting genes involved in MDR. Eid et al. also reported that* Fallopia japonica* (FJ) could modulate the function of ABC drug transporters to overcome multidrug resistance in cancer cells [130].

5-Fluorouracil (5-FU), a common chemotherapeutic agent used for tumor treatment, by itself has inadequate response rates, highlighting the need for improving the effects for these patients [131–134]. Baicalein, a flavonoid derived from the root of* Scutellaria baicalensis*, was reported to increase the sensitivity of AGS cells to 5-FU treatment under hypoxia. In addition, the hypoxia-enhanced glycolytic flux and expression of several critical glycolysis-associated enzymes (HK2, LDH-A, and PDK1) in the AGS cells were suppressed by baicalein. These findings suggested that inhibition of glycolysis via regulation of the PTEN/Akt/HIF-1*α* signaling pathway might be one of the mechanisms whereby baicalein reverses 5-FU resistance in cancer cells under hypoxia [131].

Sorafenib, a standard first-line therapeutic treatment for patients with advanced hepatocellular carcinoma (HCC), is also demonstrated to be hampered by the development of drug resistance in recent years [134, 135]. The activation of Akt by sorafenib was thought to be responsible for this resistance [136]. It was found that Bufalin, which is the major active ingredient of the traditional Chinese medicine Chan Su, could inhibit Akt activation and reverse drug resistance to sorafenib. Further studies reported that bufalin reversed acquired resistance to sorafenib by downregulating phosphorylated Akt in an ER-stress-dependent manner via the inositol-requiring enzyme 1 (IRE1) pathway [137].

### 3.5. TCM Attenuates Oncogenicity of CSCs

Cancer stem cells (CSCs) existing in the tumor play a crucial role in carcinogenesis though in a very few quantity [138]. Julius Cohnheim once inferred that tumors might arise from stem cells left over from embryonic development [139]. Recently, researches have further demonstrated that CSCs had the ability of self-renewing, invasion, metastasis, immunosuppressive, and multidrug resistance. Hence, CSCs are proposed to be the main cause of cancer relapse after resisting several therapies [140]. Evidences showed that TCM attenuated oncogenicity of CSCs. When treated with bufalin, the sphere of CSCs could not get attached to the flask and failed to differentiate, which was indicated by the stable expression of stem cell marker CD133 and OCT-4 in the condition permissive to differentiation. Treatment of bufalin also suppressed the single cells isolated from the sphere to form sphere again in the nonadhesive culture system, and a decreased expression of proliferation marker Ki67 was also detected in these cells [141]. Besides, CSCs tumor spheres lowly expressed Fas and highly expressed membrane complement regulatory proteins and Foxp3, which were associated with a high frequency of metastasis [142]. Some medicinal herbs had been proved to reverse CSCs immune suppression and suppress CSCs metastasis via dual-blocking epithelial-mesenchymal transition (EMT) and CSCs properties [143]. Pien Tze Huang (PZH), a well-known and ancient TCM formula, was reported as an operative medicine that significantly and does-dependently inhibited the viability and promoted the apoptosis and differentiation of the colorectal CSCs via suppressing the Notch1 pathway. Studies also revealed that PZH markedly inhibited the mRNA levels of ABCB1 and ABCG2, which are members of the ATP-binding cassette (ABC) transporter superfamily, thereby contributing to the side population phenotype and multidrug resistance [144].

## 4. Summary

The mutual and interdependent interaction between tumor and its microenvironment is a crucial topic in cancer research. Previously, we have made headways in understanding and preventing tumors, but most of them are focused only on cancer cells. Recently, accumulated data indicates that TME played a crucial role in protecting cancer cells [145]. Studies declare that tumor microenvironment consists of cells, soluble factors, signaling molecules, extracellular matrix, and mechanical cues that can promote neoplastic transformation, support tumor growth and invasion, protect the tumor from host immunity, foster therapeutic resistance, and provide niches for dormant metastases to thrive [145]. Thus, multiple abnormal segments have existed in malignant diseases. The synergistic interplay between tumor cells and microenvironment plays a key role in the progress of cancer, for which can offset the actions of soloes target for drugs [146]. So it has become a new trend for researchers to block the interplay between tumor and microenvironment via comprehensive treatments and combined application of drugs.

A better understanding of interplay between tumor cells and microenvironment may be a crucial key to improve therapeutic efficacy. For example, it was well known that radiotherapy directly caused cancer cells death through the induction of DNA damage. In the meantime irradiated tumors stimulated the immune system and caused immunogenic cell death by releasing tumor antigens and damage-associated molecular patterns, which promotes the uptake of dying cells and triggers a cytotoxic T-lymphocyte response [147, 148]. Zegers et al. finally demonstrated that radiotherapy combined with the immune cytokine L19-IL2 could provide long-lasting antitumor effects [148]. Therefore, compared with antitumor immune response that radiotherapy provides alone, the addition of active immunotherapy may increase the therapeutic potential. In addition, it was reported that dying tumor cells through the apoptosis generated potent growth-stimulating signals to stimulate the repopulation of tumors undergoing radiotherapy. And activated caspase 3, a key executioner in apoptosis, was also involved in the growth stimulation [149].

**Figure 1:**
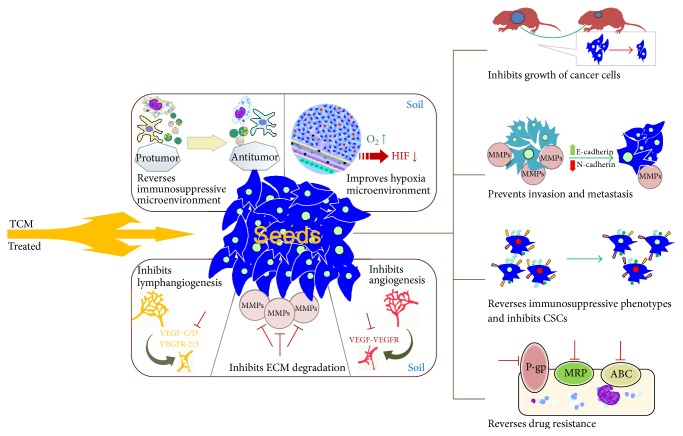
The confirmed mechanism of TCM on tumor cells and microenvironment. TCM can inhibit “seeds” and regulate “soil” to suppress tumor development and recurrence via diverse ways. The mechanism of TCM inhibiting tumor “seeds” includes inhibiting growth, invasion, metastasis of cancer cells, and reversing immunosuppressive phenotypes and drug resistance. Meanwhile, TCM regulates “soil” through remodeling immunosuppressive microenvironment, hypoxia microenvironment, and angiogenesis/lymphangiogenesis, and ECM are also reversed when treated by TCM.

When targeted, cancer cells alter the surrounding tumor microenvironment to protect themselves. Although studies showed that TCM inhibit the cancer cell growth and tumor microenvironment through various mechanisms (Figure 1), the interplay between the tumor cells and microenvironment is not clear. By understanding the mechanisms by which TCM inhibits tumor cells and microenvironment, novel cancer therapeutics can be evolved.

## Figures and Tables

**Table 1:** Several clinical studies have confirmed the effectiveness of TCM in cancer treatment.

Drug	Type of cancer	Phase	Method	Interventions		Outcomes	Cite
				Control group	Treatment group		
Ginsenoside Rg3	NSCLC	Clinical stages II/IIIa, postoperative patients	RCT, not blinded 4–6 cycles	Chemotherapy (NP/CE/GP)	Shenyi capsule, or it combines with chemotherapy	Improves the life span of patients	[7]

Cantharidinate	NSCLC	Middle-late stage	RCT, not blinded 4 cycles	GP	Combines with cantharidinate	Improves clinical effects and life quality, lowers the toxic/adverse effects of chemotherapy	[8]

Astragalus polysaccharide	NSCLC	Clinical stages IIIB or IV advanced disease	RCT, not blinded 3 cycles	Vinorelbine and cisplatin	Combines with APS	Improves patients' QOL	[9]

Xiaoaiping	Breast cancer	Neoadjuvant chemotherapy	RCT, not blinded duration, 12 weeks	TEC neoadjuvant chemotherapy	Combines with Xiaoaiping injection	Significantly enhances short-term and long-term efficacies of neoadjuvant chemotherapy	[10]

Atractylenolide I	Gastric cancer	Cachexia patients	RCT, not blinded duration, 7 weeks	Nutritional supplementation	Adds atractylenolide I	Improves appetite and KPS status and decreases PIF positive rate	[11]

Jianpi Huayu therapy	Hepatocellular carcinoma	Firstly treated patients	RCT, not blinded duration, 1 year	Hepatectomy and conventional western medicine treatment	Combines with Jianpi Huayu therapy	Reduces postoperative recurrence and metastasis, improves DFS and OS	[12]

Huachansu	Gallbladder carcinoma	Locally advanced or metastatic	Not RCT, not blinded, continued until termination events	Gemcitabine-oxaliplatin	Combines with Huachansu injection	Makes chemotherapy well tolerated and improves the QOL of patients	[13]

Curcumin	Colorectal cancer	Liver metastases	Escalation trial, 12 cycles of chemotherapy	FOLFOX chemotherapy	Combines with curcumin	Prolongs median PFS and improves treatment response	[14]

Compound Zhebei granules	Acute leukemia	Posttreatment and defined as refractory	RCT, double-blind and multicentral concurrent control, 14 days	Multiple chemotherapeutic schemes	Combines with compound Zhebei granules	Increases the clinical remission rate	[15]

**Table 2:** Different mechanisms and channels of TCM inhibiting the growth of tumor cells.

Cancer	Drug	Main mechanisms	Main channels	Related biomarkers	Cite
Lung cancer (A549 Cells)	Xiaoji decoction	Inhibits proliferation and induces apoptosis	Signaling Akt pathway	BAD ↑, caspase-9 ↑	[94]

Lung cancer (A549 Cells)	Oxymatrine	Induces apoptosis	Bcl-2 family	Bax ↑ and Bcl-2 ↓	[88]

Esophageal carcinoma (CaEs-17)	Soups of *Rosa roxburghii* Tratt and *Fagopyrum cymosum*	Inhibits proliferation and induces apoptosis	Ki-67, Bcl-2 family	Bax ↑ and Ki-67, Bcl-2 ↓	[92]

Esophageal carcinoma (KYSE150, Eca-109)	*Marsdenia tenacissima* extract	Inhibits proliferation	MAPK signaling pathway	cyclinD1, p-ERK ↓	[84]

Gastric cancer (SGC-7901)	Sanpi Pingwei formula	Inhibits proliferation and induces apoptosis	Bax, p53, and Bcl-2	Bax, p53 ↑ and Bcl-2 ↓	[90]

Gastric cancer (AGS, MGC803)	Arsenic sulfide	Induces apoptosis	p53	Bax, MDM2 ↑ and Bcl-2 ↓	[96]

Colorectal cancer (SW480)	Jianpi Huayu decoction	Inhibits proliferation and induces apoptosis	G0/G1-phase cell cycle arrest, caspase-cascade activation and execution	p27 ↑ and cyclinD1, cyclinD2, cyclinD3, cyclinE1, CDK4, CDK6, CDK2 ↓	[95]

Colorectal cancer (LOVO)	Emodin	Induces apoptosis	Bcl-2 family	Bax ↑ and Bcl-2 ↓	[85]

Live cancer (Huh7, Hep3B, HA22T)	Bufalin	Inhibits proliferation and triggers autophagy	G2/M phase arrest and JNK pathway	TNF, BECN-1, MAPK, ATG8 ↑ and Bcl-2, Bid ↓	[81]

Breast cancer (MCF-7, MDA-MB-231)	San-Zhong-Kui-Jian-Tang	Inhibits proliferation and induces apoptosis	p21/WAF1 levels, p53, Bcl-2 family	p21, Bax, Bak ↑ and cyclinD1, cyclinD2, Bcl-2 ↓	[93]

Ovarian cancer (OVCAR-3)	Pien Tze Huang	Inhibits proliferation	AKT-mTOR pathway	AKT, (p)-AKT, mTOR p-mTOR proteins, CDK4, CDK6 ↓	[79]
